# Emergence of life: Physical chemistry changes the paradigm

**DOI:** 10.1186/s13062-015-0060-y

**Published:** 2015-06-10

**Authors:** Jan Spitzer, Gary J. Pielak, Bert Poolman

**Affiliations:** R&D Department, Mallard Creek Polymers, Inc., 2800 Morehead Rd, Charlotte, NC 28262 USA; Department of Chemistry, Department of Biochemistry and Biophysics and Lineberger Comprehensive Cancer Center, University of North Carolina at Chapel Hill, Chapel Hill, NC 27599 USA; Department of Biochemistry, Groningen Biomolecular Sciences and Biotechnology Institute & Zernike Institute for Advanced Materials, University of Groningen, Nijenborgh 4, 9747 AG Groningen, The Netherlands

**Keywords:** Chemical evolution, Darwinian evolution, Origin of life, Diurnal gradients, Chemical complexity, Biomacromolecular crowding, Non-covalent intermolecular forces, Molecular recognition, Cellular organization

## Abstract

Origin of life research has been slow to advance not only because of its complex evolutionary nature (Franklin Harold: In Search of Cell History, 2014) but also because of the lack of agreement on fundamental concepts, including the question of ‘what is life?’. To re-energize the research and define a new experimental paradigm, we advance four premises to better understand the physicochemical complexities of life’s emergence:

1. Chemical and Darwinian (biological) evolutions are distinct, but become continuous with the appearance of heredity.
2. Earth’s chemical evolution is driven by energies of cycling (diurnal) disequilibria and by energies of hydrothermal vents.
3. Earth’s overall chemical complexity must be high at the origin of life for a subset of (complex) chemicals to phase separate and evolve into living states.
4. Macromolecular crowding in aqueous electrolytes under confined conditions enables evolution of molecular recognition and cellular self-organization.

We discuss these premises in relation to current ‘constructive’ (non-evolutionary) paradigm of origins research – the process of complexification of chemical matter ‘from the simple to the complex’. This paradigm artificially avoids planetary chemical complexity and the natural tendency of molecular compositions toward maximum disorder embodied in the second law of thermodynamics. Our four premises suggest an empirical program of experiments involving *complex* chemical compositions under *cycling gradients* of temperature, water activity and electromagnetic radiation.

## Introduction: the problem

*“The study of dynamic self-assembly is in its infancy”* [1]

The tension between Darwin’s ‘*…some one primordial form into which life was first breathed*’ and Pasteur’s ‘*all life from life*’ defines one of the least understood natural phenomena – the emergence of living states of matter [2, 3]. Even though many scenarios for emergence have been developed, they are controversial and unresolved. They have been characterized as ‘big questions, big problems’ [4]. Extensive literature on life’s origins is collected and critically annotated in “Origins of life – the Central Concepts” [5]. More recent advances, including historical, philosophical and evolutionary aspects, are also available [6–8]. A long but non-exhaustive list of books on the origin of life is given in Table 1.

**Table 1:** This chronological list of *books only* shows that the literature on ‘origins’ has grown tremendously in the last 20 years

Author	Year	Title	Publisher
Bernal JD	1967	The Origin of Life	The World Publishing Co, Cleveland & New York
Kenyon DH and Steinman G	1969	Biochemical Predestination	McGraw-Hill Book Co, New York
Fox SW and Dose K	1972	Molecular Evolution and the Origin of Life	WH Freeman, San Francisco
Jacob F	1973	The Logic of Life. A History of Heredity	Pantheon Books, New York
Miller SL and Orgel LE	1974	The Origins of Life on the Earth	Prentice-Hall, Englewood Cliffs NJ
Crick F	1981	Life Itself. Its Origin and Nature	Simon & Schuster, New York
Cairns-Smith AG	1985	Seven Clues to the Origin of Life: a Scientific Detective Story	Cambridge University Press, Cambridge
Shapiro, 1986	1986	Origins. A Skeptic's Guide to the Creation of Life on Earth	Simon & Schuster, New York
Eigen M	1992	Steps toward life	Oxford University Press, Oxford
Morowitz HJ	1992	Beginnings of Cellular Life	Yale University Press, New Haven CT
Deamer DW and Fleischaker GR	1994	Origins of Life. The Central Concepts	Jones & Bartlett Publishers, Boston
de Duve C	1995	Vital Dust: Life as a Cosmic Imperative	Basic Books, New York
Kaufmann S	1995	At Home in the Universe. The Search for the Laws of Self-Organization and Complexity	Oxford University Press, New York
Zukerman B and Hart MH (eds)	1995	Extraterrestials: Where Are They?	Cambridge University Press, Cambridge
Zubay, G	1996	Origins of Life on the Earth and in the Cosmos	Wm. C. Brown Publishers, Dubuque IA
Brack A (ed)	1997	The Molecular Origins of Life. Assembling Pieces of the Puzzle	Cambridge University Press, Cambridge
Margulis L and Sagan D	1997	Microcosmos. Four Billion Years of Microbial Evolution	University of California Press, Berkeley
Davies P	1999	The Fifth Miracle. The Search for the Origin and Meaning of Life	Simon & Schuster, New York
Dyson F	1999	Origins of Life	Cambridge University Press, Cambridge
Fry I	1999	The Emergence of Life on Earth. A Historical and Scientific Overview	Rutgers University Press, New Brunswick NJ
Lahav N	1999	Biogenesis. Theories of Life's Origin	Oxford University Press, Oxford
Smith JM and Szathmary E	1999	The Origins of Life. From the Birth of Life to the Origins of Language	Oxford University Press, Oxford
Willis C and Bada J	2000	The Spark of Life: Darwin and the Primeval Soup	Perseus Publishing, Cambridge MA
Schopf JW (ed)	2002	Life's Origin. The Beginning of Biological Evolution	University of California Press, Berkeley
Ganti T	2003	The Principles of Life	Oxford University Press, New York
Gilmore I and Sephton MA	2004	An Introduction to Astrobiology	Cambridge University Press
Knoll AH	2003	Life on a Young Planet. The First Three Billion Years of Evolution on Earth	Princeton University Press, Princeton
Hazen RM	2004	Genesis. The Scientific Quest for Life's Origin	Joseph Henry Press, Washington DC
Mayr E	2004	What Makes Biology Unique?	Cambridge University Press, Cambridge
Luisi PL	2006	The Emergence of Life: from Chemical Origins to Synthetic Biology	Cambridge University Press, Cambridge
Sullivan WT III and Baross JA (eds)	2007	Planets and Life. The Emerging Science of Astrobiology	Cambridge University Press, Cambridge
Barrow JD, Morris SC, Freeland SJ, Harper, Jr. CL (eds.)	2007	Fitness of the Cosmos for Life	Cambridge University Press, Cambridge
Benner S	2008	Life, the Universe…and the Scientific Methods	The FfAME Press, Gainsville FL
Rasmussen S et al. (eds)	2008	Protocells: Bridging Non-living and Living Matter	MIT Press, Cambridge MA
Bedau MA and Cleland CE	2010	The Nature of Life. Classical and Contemporary Perspectives from Philosophy and Science	Cambridge University Press, Cambridge
Yarus M	2010	Life from an RNA World. The Ancestor Within	Harvard University Press, Cambridge MA
Atkins P	2011	On Being. A Scientist's Exploration of the Great Questions of Existence	Oxford University Press, Oxford
Deamer DW	2011	First Life. Discovering the Connections between Stars, Cells, and How Life Began	University of California Press, Berkeley
Impey C	2011	The Living Cosmos. Our Search for Life in the Universe	Cambridge University Press, Cambridge
Koonin EV	2011	The Logic of Chance: The Nature and Origin of Biological Evolution	FT Press Science
Rutherford A	2013	Creation: the Origin of Life & the Future of life	Penguin, London UK
Pross A	2014	What is Life? How Chemistry Becomes Biology	Oxford University Press Oxford UK
Harold FM	2014	In Search of Cell History. The Evolution of Life's Building Blocks	The University of Chicago Press, Chicago
Lane N	2015	The Vital Question. Energy, Evolution, and the Origins of Complex Life	W.W. Norton, to be published

The origins research is based on the Oparin-Haldane hypothesis (promulgated in 1924 and 1929) that only natural, ultimately knowable processes brought about ‘life as we know it’ [3]. Oparin’s and Haldane’s papers are included in the first book in the table by J. D. Bernal: “Origin of Life”

The fundamental question is whether ‘life as we know it’ – its astonishing chemical complexity – is a very improbable occurrence or a nearly inevitable evolution of dynamic off-equilibrium chemical matter. The answers have been categorized [9, 10] into two ‘camps’– the camp of ‘almost miracles’ [11–13] and the camp of the inevitability of laws of physics and chemistry [3, 14]. One question in the law camp is whether untangling the complexity of life’s emergence requires new, as yet unknown molecular principles, for example autocatalytic ‘hypercycles of replicators’ [15], ‘metabolic networks’ of varying complexity and dimensionality [16–19], ‘molecular ecology of composomes’ [20], ‘dynamic kinetic stability’ [21] and others [5–7, 22].

We propose a less conjectural approach, based on established facts and laws that we exploit to frame workable premises for origins research [3, 23, 24].

### The premises

Our goal is to secure a sound physicochemical foundation for *origins biology* in terms of *evolutionary chemistry* (1). We postulate premises that derive from accepted (‘textbook’) physicochemical laws that govern behavior of ‘inanimate’ molecules, such as Brownian motion [25], and from relevant facts of molecular microbiology and planetary sciences. We do not ascribe biological properties to molecules, such as self-replication or the principles of Darwinian evolution, e.g., natural selection.

**Box 1.** Explanations of some chemical, planetary and evolutionary terms

**Fig. 1:**
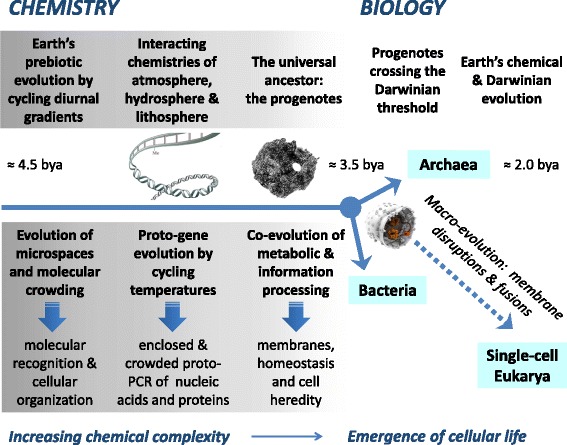
Earth’s physicochemical evolution from 4.5 bya (billion years ago): the continuity of chemistry and biology. Earth’s chemistry evolved in atmosphere, hydrosphere and lithosphere with rampant phase separations of microspaces (0.1 - 1000 μm) driven by diurnal cycles of solar radiation, temperature and water activity

**Evolutionary chemistry.** In the context of life’s emergence, evolutionary chemistry deals with non-equilibrium inanimate matter driven by cyclic physicochemical gradients in open thermodynamic systems. The most important gradients are cyclic diurnal (day-and-night) gradients of solar radiation, which bring about cyclic temperature and water activity gradients (hydration/dehydration cycles) in local Earth’s environments. (In chemical engineering, evolutionary chemical processes occur during the start-up of large continuous chemical reactors, when the reactor output is not constant but evolves towards the desired steady state [85]).
**Open thermodynamic system.** Earth, as a chemical reactive system, exchanges matter and energy with the surroundings (cosmos), is an open system, the boundary being determined by gravitation. Chemical reactors represent another example, with different boundaries.
**Diurnal disequilibria.** Cyclic physicochemical disequilibria, *e.g.* of temperature and water activity and hence also of chemical potentials of dissolved molecules (via Gibbs-Duhem equation), brought about by day and night changes of solar electromagnetic radiation *in local regions* of rotating Earth’s surface. Polymeric substances considerably complicate the physical chemistry of multicomponent and multiphase systems because second order transitions, such as glass temperature- or sol–gel transitions.
**Hydrothermal vents.** Discovered in 1977 at the bottom of the Pacific Ocean, hydrothermal vents are ‘hot springs’ where tectonic plates are moving apart, allowing hot magma to rise and superheat seawater, which then escapes as hot springs or even geysers. The superheated water dissolves many minerals that precipitate and form tall porous ‘chimneys’ and other structures through which hot water containing precipitating minerals (*e.g.* black sulfides) escapes as it mixes with cold seawater. There are various kinds of vents, with different mineral content (colors), temperatures, and rates of flow. Bacteria with different metabolisms (redox chemistries) live in biofilms around the vents and provide food for unique eukaryotic organisms [36]. A unique example is “Lost City” in the Atlantic Ocean, which contains vents of CaCO_3_ that release methane and hydrogen in very alkaline seawater, where bacteria can oxidize methane [48].
**Hadean Earth.** The first eon of Earth’s history that lasted from about 4.5 to 3.5 billion years ago as determined by radioactive dating of ancient rocks. Fossilized ‘biofilms’ were dated at about 3.5 billion years, when the Hadean eon ends and the Archaean eon begins.
**Progenotes, the universal ancestor and LUCAs.** Woese’s *phylogenetic* concept representing ‘living states’ with fixed genetic code but with transcription/translation machineries still evolving. The genes undergo extensive horizontal gene transfer, which prevents the appearance of vertical heredity (lineages, ancestry). The necessary environmental conditions for the appearance of progenotes and their *physicochemical* characteristics were not considered, particularly the necessity for enclosures (proto-membranes), which are mandated by the 2^nd^ law of thermodynamics, Fig. 1. The progenotes practiced different chemistries in respect to exploiting environmental chemicals and energies, particularly in respect to gaining energy from membrane-localized oxidations and reductions. They were chemically evolving toward the Darwinian thresholds from which Last Universal Common Ancestors (LUCAs) emerged as modern cells. There may have been only one LUCA, as speculated by Darwin *‘…one primordial form’*, but likely there were many more LUCAs (now likely extinct), from which the three domains of life emerged: Bacteria, Archaea and Eukaryota; all three have been coevolving with plasmids, phages, and viruses, and are still undergoing horizontal gene transfer as documented phylogenetically [86–89].
**Darwinian threshold.** Woese’s phylogenetic concept that represents an evolutionary period during which expression of genes and their transcription and translation become stabilized, thus enabling the transition to modern cells with vertical heredity – the progenotes became ‘genotes’ [29, 30]. From a physicochemical standpoint, during this period proto-nucleoids with proteinaceous lipid-like (surfactant) bilayers evolved into a ‘gelled’ and mechanically stronger cellular scaffold [41, 46], which enabled cellular homeostasis and diminished horizontal gene transfer sufficiently for vertical heredity to persist (the beginning of biology).
**Micro-evolution (physicochemical mechanism).** Heritable errors in the genome of one cell, *i.e.* DNA chemical changes caused by fluctuating and changing environments resulting in imperfect copying processes; the ‘errors’ range from point mutations to gene duplications and gene loss but do not involve direct membrane disruptions with ‘foreign’ membranes and foreign environmental DNA. Microevolution represents gradual acquisition of new capabilities to survive, as envisaged originally by Darwin.
**Macro evolution (physicochemical mechanism).** Heritable errors in the genome involving plasma membrane breakage and fusions with other membranes and environmental DNA not related to the normal course of cell division, allowing ‘foreign’ DNA to enter the cell (horizontal gene transfer). These are large scale ‘errors’ sometimes denoted as evolutionary *saltations*. Normally they are fatal but when successful – extremely rarely – a structurally new kind of cell emerges with unique capabilities (innovations) very quickly – essentially leapfrogging microevolution; eukaryotic cells likely originated by such a mechanism (fusion of two bacterial cells). Eukaryotic cells and multicellular organisms were then evolving by interactions of their nuclear membranes with the membranes of other organelles, and with plasma membranes of other cells. Such membrane reorganizations underlie horizontal gene transfer, natural bacterial competence and endosymbiosis between many kinds of organisms, as well as the protocols of genetic engineering and of reconstitution of (membrane) proteins in vesicles.
**Homeostasis.** In the context of life’s emergence, homeostasis represents sufficient proto-biochemical and structural integrity for progenotes (or LUCAs) to withstand reasonably large changes in physicochemical environments, particularly of temperature and water activity, as they evolve across the Darwinian threshold.
**Proto-heterotrophy.** Heterotrophic carbon sources originate from ‘dead’ organic matter, as contrasted with autotrophic carbon sources, of which the most prevalent is ‘inorganic’ carbon dioxide. In the context of life’s emergence, progenotes and LUCAs could obtain carbon from proto-biomolecules of ‘dead’ progenotes and LUCAs, *i.e.* proto-heterotrophically, as different carbon compounds became available during the evolutionary time of progenotes crossing the Darwinian threshold. Such very early heterotrophy tremendously accelerated chemical evolution of progenotes into LUCAs and ‘life as we know it’. Remarkably, some extant bacteria can utilize tholins as the only source of carbon [68]. Tholins are complex carbon compounds resembling molecules of coal or tar sands, presumably occurring on Saturn’s moon Titan.

Our premises identify physicochemical conditions for the emergence of life, and suggest new experiments with gradients of electromagnetic radiation, temperature and water activity that keep *complex chemical mixtures* in cyclic disequilibria, i.e., ‘repeatedly stoked with energy’, and hence in continuous physicochemical evolution [26]. The premises are introduced below and discussed in terms of both chemical and biological paradigms of origins research.

#### Chemical evolution and Darwinian evolution are distinct but become continuous through the Darwinian threshold

In acknowledging the autonomy of large parts of biological sciences that cannot be readily ‘reduced’ to chemistry, such as animal behavior or ecology [27], living microbial cells represent a tangible realization of chemical evolution from inanimate matter to cellular life, as shown in Fig. 1. This evolutionary relationship suggests that Dobzhansky’s dictum (‘nothing in biology makes sense except in the light of [Darwinian] evolution’) can be applied to origins of biology, and paraphrased as ‘nothing in the origin of life makes sense except in the light of *chemical* evolution’. This view anchors inanimate chemical evolution to microbial cells *and* to their Darwinian evolution by adopting the phylogenetic concepts of the universal ancestor (a community of progenotes) and the Darwinian threshold, which gave rise to the three domains of life – Bacteria, Archaea and Eukaryota [28–31]. Although Earth’s prebiotic evolutionary chemistry can be studied indirectly [32], the first premise also points to contemporary experiments with complex ‘biotic’ mixtures obtained from dead populations of microorganisms [33, 34]. The question then becomes how such a disordered (high entropy) proto-bacterial system can be re-organized into an evolving living state, or whether there are compelling theoretical or technical reasons why this is impossible.

#### Earth’s chemical evolution is driven by the energies of diurnal disequilibria and hydrothermal vents

The sources of Earth’s evolving chemical complexity are the energies of solar diurnal disequilibria [35] and the energies of physicochemical gradients at hot hydrothermal vents [36]. The diurnal disequilibria arise naturally when Earth’s rotation converts ‘constant’ solar radiation into cyclic energy gradients that drive chemical reactions at Earth’s oceanic and rocky surfaces; hydrothermal vents release metallic ions and other compounds into the ocean, enriching the ocean’s molecular complexity. Different chemistries driven by solar radiation and by hydrothermal vents interact, particularly at the three-phase boundary of irradiated tidal seashores, and thus further increase the chances of life’s emergence. Thus, the evolution of primordial chemical compositions and processes was initially driven by Earth’s diurnal cycles that caused repeated colloidal phase separations – the appearances and disappearances of ‘first microspaces’ [26] (Fig. 1). Today’s microbial cell cycle can be viewed as an ‘evolutionary echo’ from the cyclic chemical disequilibria of the rotating surface of Hadean Earth (Box 1). The cell cycle had (partially) decoupled from physicochemical diurnal gradients when the stabilization of lipid-protein membranes and information processing allowed cell heredity to take root [2].

#### Earth’s chemical complexity must be high for a subset of chemicals to evolve into living states

This complexity arises from the totality of all reacting chemical compounds, only *a subset* of which can separate into microspaces with permeable boundaries – the ‘enclosures of future cells’ [26, 32]. The higher the physicochemical diversity of Earth’s environments, the higher the chemical complexity of the separated microspaces, and the higher the chance of living states evolving from them. The micro-spaces, being *non-equilibrium open* thermodynamic systems (i.e., matter and energy can be transferred through the boundaries), chemically evolve toward living states, while the environments outside become chemical pools of nutrients. At the biological end of the chemical evolutionary continuum, microbial cells can also be viewed as (self-constructing) chemically evolving open thermodynamic systems that exchange materials and energy with their environments as they grow and divide. One way to define chemical complexity is to enumerate all different molecules and their concentrations. Here ‘molecules’ are broadly defined to include macromolecules, ions and polyelectrolytes. Usual methods of physical chemistry to express concentrations (chemical potentials) become impractical because bacterial cells contain thousands of different molecules at high total volume fractions. Instead, cellular chemical complexity has been characterized by the term ‘crowding’ resulting from the high total volume fraction of all molecules [37], some of which may be unknown or individually at low concentrations, which leads to our last premise.

#### Macromolecular crowding in aqueous electrolytes enables evolution of molecular recognition and cellular self-organization

**Fig. 2:**
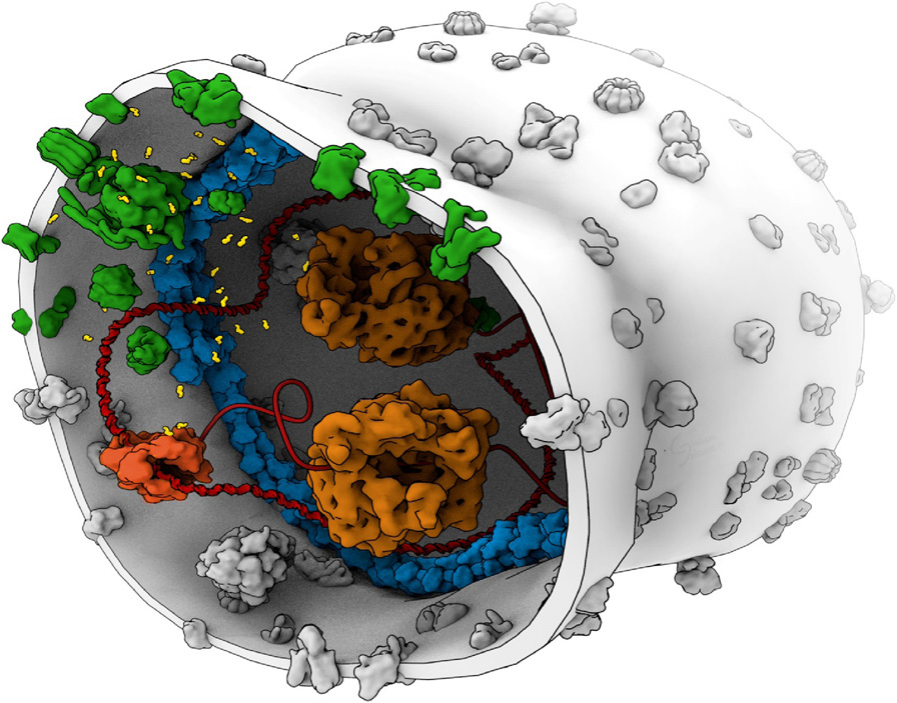
A cartoon of a bacterial cell intentionally shown ‘uncrowded’ to highlight the key living processes: metabolism (green, cytoplasmic and membrane proteins), information processing (red/orange, DNA and RNA and transcription and translation machineries), and reproduction (blue, membrane morphogenesis and division)

**Fig. 3:**
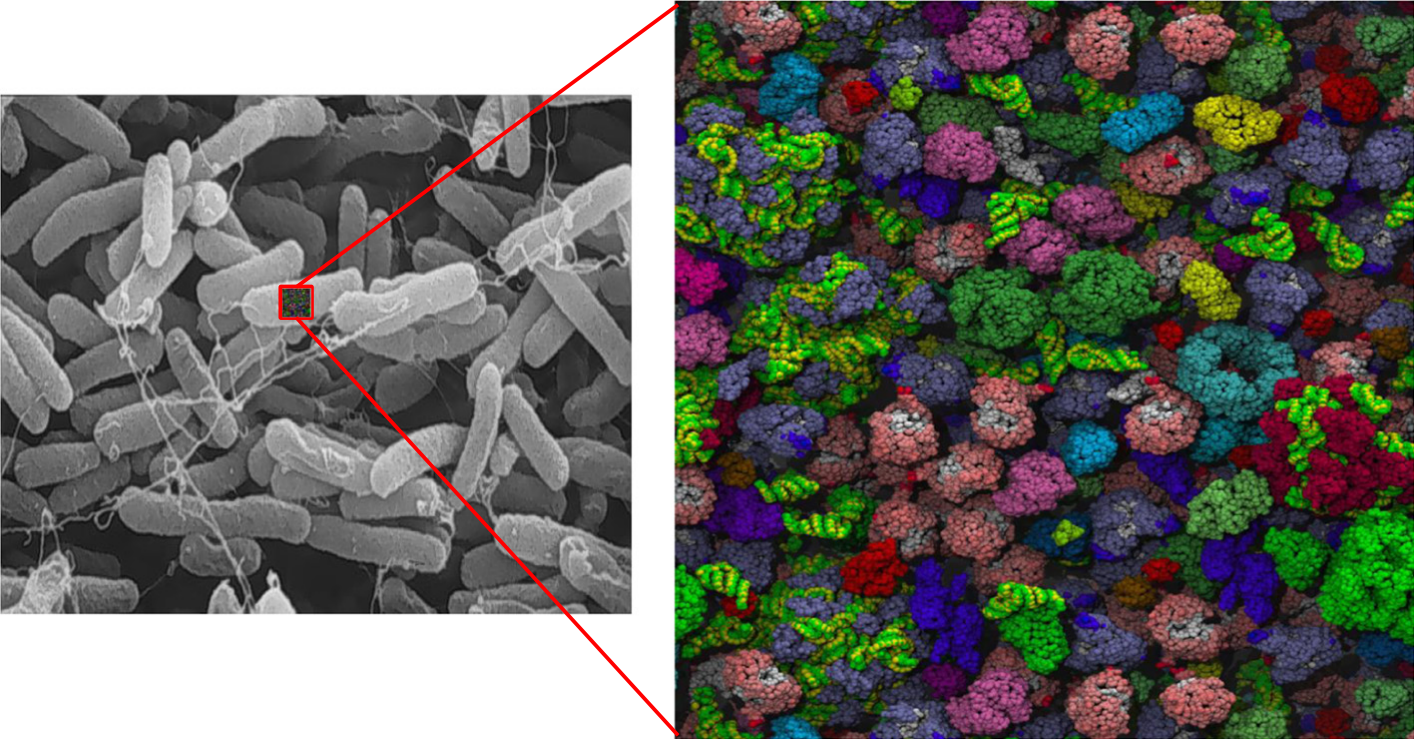
Left: *Escherichia coli cells* as rods about 1000 nm in diameter and 3000 nm long. Right: computer simulation of crowding in an *E. coli* cell; proteins/RNA occupy ~25 % of cell volume; if not in complexes, proteins are on average about 1–2 nm apart. There are about 4 × 10^6^ proteins/cell, 20 × 10^9^ H_2_O/cell, and 12 × 10^7^ ions/cell, mostly K^+^, corresponding to ionic strength of about 0.25 M

It is a fact that all living cells are crowded in an aqueous solution of mixed salts – that is, in an electrolyte, cf*.* Figs. 2 and 3 for the case of bacteria [38–41]. The evolutionarily ‘first’ bacterial cells are somewhat more crowded than eukaryotic cells, and some of them can function successfully at very high crowding levels under hyperosmotic conditions [42, 43]. Cellular biomacromolecules are crowded in aqueous electrolytes of high ionic strength (>0.1 M), cf*.* Figs. 2 and 3, which makes screened electrostatic forces act over distances on the order of about one nanometer – the Debye length [26, 44, 45]. Thus, three non-covalent interactions: the hard core excluded volume effect of biomacromolecular crowding (the distances between inert biomacromolecular surfaces), hydration (2 to 4 water molecules) and screened electrostatic forces act over a *commensurate* distance of just below one nanometer. Furthermore, this distance depends only weakly on temperature over the liquid range of water, which allows life to evolve both in superheated water and in ice-water mixtures [26].

Molecular recognition and cellular self-organization cannot evolve in unconfined spaces, i.e., in primordial soups or at surfaces, because of the disordering effects of the second law of thermodynamics. Recognition and self-organization cannot evolve also in *uncrowded* enclosed systems [46], where molecular surfaces are far from each other. In such dilute systems, the thermal disordering effects of the second law overcome the ordering effects of attractive intermolecular forces (a precursor phenomenon to phase separation, for example the formation of micro-gels and other colloidal nano-phases). Hence under uncrowded conditions, non-covalent molecular forces fail to maintain functional cellular organization. Orgel’s dictum expresses this premise succinctly – ‘molecules that stay together, evolve together’ [47].

## Discussion

The evolutionary continuity between chemistry and biology, our first premise, is uncontroversial: complex non-equilibrium chemical matter inevitably evolves such that under some conditions the emergence of living states becomes imperative, eliminating the discontinuous (‘miraculous’) mechanism of ‘life being breathed’ into inanimate matter by external experimenters. This premise adopts Woese’s *phylogenetic* concepts (Fig. 1) of: (i) The Universal Ancestor – the roots of the Tree of Life represented by a ‘biofilm’ (community) of progenotes with evolved (fixed?) genetic code but still evolving transcription and translation machineries with unstable proto-membranes that allowed extensive horizontal gene transfer; (ii) the Darwinian Threshold, when proto-membranes stabilized sufficiently to diminish horizontal gene transfer and allow the appearance of vertical heredity and hence of biological species. We note that Woese did not specify the physicochemical mechanism of the Darwinian threshold; we suggest that it included an increased mechanical stabilization of membranes and the coupling of DNA replication and protein synthesis with membrane growth. This mechanism implies that nucleic acids, proteins and membranes coevolved in complex *crowded* proto-biofilms before single cells assumed independent existence, cf*.* Fig. 1. Ancestral bacteria were likely the first to cross the Darwinian threshold and separate as individual cells [29, 30].

With the appearance of three cellular designs – bacterial, archaeal and eukaryotic, Darwinian evolutionary processes then progressed by (i) micro-evolutionary errors in the genome, i.e., DNA chemical changes, and by (ii) macro-evolutionary (‘saltational’) rare events involving interactions of different cells and the morphogeneses of their membranes (Box 1). For example, eukaryotic cells likely arose by direct cellular interactions – membrane fusions under repeated hydrating and dehydrating conditions of cycling temperatures cf*.* Fig. 1. In both cases, the resulting ‘random’ individual mutants (Darwinian variations) are tested by physicochemical and biological environments for short-term viability (‘staying alive’) and for reproductive competence leading to heredity (ancestry, lineages) on evolutionary timescales (Darwinian natural selection).

Living states are here defined at a single cell microbial level [2] because – from both physicochemical and biological standpoints – *no* molecular assemblies other than bacterial, archaeal and eukaryotic can be alive, reproduce and exhibit Darwinian evolution. Our application of physicochemical laws thus excludes the world of unconfined (at surfaces or in solutions) ‘replicator molecules’ as a plausible stage in the conversion of inanimate matter into cellular life, as well as unconfined worlds of ‘self-assembling’ metabolic networks. *How could proto-biomolecules of whatever sort prevent their own molecular (entropic) diffusion and macroscopic (physical) mixing with water?*

The paradigms of molecular replicators and self-organizing metabolic networks ignore the second law of thermodynamics [24], because the presumed molecules select themselves (un-mix) from all the other prebiotic chemicals. Instead, an energy source (work) is needed for ‘un-mixing’ when it is enabled by the formation (phase separations) of microspaces. Thus, the second law of thermodynamics demands that *phase separations into microspaces*, no matter which (evolving) chemistries they involve, are a prerequisite for the beginning of chemical evolution toward cells, Fig. 1. For example, phase separations may yield inorganic ‘hatcheries of life’ at alkaline hydrothermal vents [48] or lipid-like proto-vesicles [49].

Our three remaining premises address conditions for *physicochemical evolution* toward cellular life: planetary energies driving chemical evolution (premise 2) under complex – multicomponent, multiphase, crowded, and non-equilibrium molecular conditions (premise 3), thereby enabling the evolution of molecular recognition and cellular self-organization (premise 4).

The second premise focuses on the energies needed to drive evolutionary chemistry in all the locales around the whole Earth [26, 50]. These energies are embedded in diurnal cycles of solar radiation and in the temperature and chemical potential gradients at hydrothermal vents. The idea that this energy is needed for chemical evolution – for the maintenance of disequilibrium (chemically reactive) conditions – should not cause controversy. Furthermore, energy is needed to counteract the working of the second law, i.e., the natural tendency for molecules to mix (increase entropy) and thus prevent the ‘self-assembly’ of living states (decrease entropy). A more precise term than ‘self-assembly’ has been coined by Franklin Harold [51] as ‘self-construction’, which does imply the expenditure of external energy. Importantly, it also implies *natural* self-construction by the energies of inanimate environments (contingent, unintentional, and without the guidance of external designers). We note that ‘dynamic self-assembly’ (self-construction) has hardly begun to be systematically studied in any way, especially in relation to the emergence of living states of matter [1].

Similarly, at the microbial end of the chemical evolutionary continuum, a bacterial cell in a Gedanken experiment cannot spontaneously re-assemble when all its constituent molecules are removed from each other to infinity and then allowed to come back together. In other words, living states cannot come about by spontaneous ‘self-assembly’ from their components even if such components were freely available. In equilibrium thermodynamic terms, assembling a cell from its components is not spontaneous or independent of path – of ‘how it’s done’. Therefore, the emergence of living states, whether historical (by the evolutionary chemistry of prebiotic Earth [32]) or ahistorical (from dead populations of microorganisms [33]), is not inevitable, but contingent upon non-equilibrium environments – on the kinds of their chemistries and the kinds and magnitudes of their energies, and hence also on ‘how it’s done’.

Earth’s diurnal physicochemical gradients that drive chemical evolution echo in the temperature cycling of polymerase chain reactions, in cellular circadian clocks, and more generally in the repeating processes of microbial growth and division. Cycling gradients, of temperature for example, are *not normally used* in either prebiotic synthetic organic chemistry, in culturing (growing) bacterial cells or in current paradigms of designing protocols for constructing synthetic protocells [52–56]. These experimental procedures do not reflect *natural* chemical and Darwinian evolutions (premise 1). Instead, they rely on direct manipulations by well-informed designers, for example, selecting the steps of non-enzymatic organic chemistry syntheses, or developing procedures to construct vesicles and protocells with desired properties. Thus, ‘constructive’ non-evolutionary (non-cyclic) processes cannot illuminate how environmental energies bring about continuous chemical evolution and the emergence of living states. The results of prebiotic synthetic organic chemistry will, however, inform the selection of initial conditions in continuous evolutionary experiments with cycling physiochemical gradients. Indeed, new research conducted with cyclic hydration/dehydration transitions in confined lamellar nano-structures shows the possibility of creating RNA-like polymers [57].

The third premise of required *chemical complexity* is physicochemically and logically straightforward [26, 41], but the least understood. The higher the complexity of evolving inanimate chemical mixtures, either prebiotic or biotic e.g.*,* dead cells, the more likely *a subset* of chemicals can separate and evolve into living states. In other words, the complexity of the whole planetary environment must be considered. The atomic and molecular diversity of the environment *must be very high at the origin of life*, and the highest complexity currently conceivable involves the interactions of non-equilibrium chemistries of hydrothermal vents with the chemistries of solar diurnal cycles (premise 2). Planets lacking sufficient quantities of key elements, particularly metals (e.g., Fe, Mo, K, Na, Mg, Ca) may not be conducive for life’s emergence even in an abundance of the usual ‘suspects’ – the biomolecules made up of C, H, O, N, S, and P evolving, for example, from prebiotic formamide chemistry [58]. Therefore, ‘life as we know it’ could not have emerged without hydrothermal vents and related geochemistry interacting with the cyclic reception of solar radiation. In that sense, Earth may be a rare planet with the ‘right’ geophysics and geochemistry, containing *all the elements* of the periodic table, and in proportions conducive for cellular emergence of life.

According to this interpretation of chemical complexity as a nominally extensive property of evolving matter, the universal (cosmic) emergence of life progresses from *the large and complex* – gravitational and nuclear evolution of stars providing chemical elements, molecules and macromolecules in planetary disequilibria – to *the small and complex*, the evolutionary chemistry of phase-separated micron-sized microspaces.

The principle of high chemical complexity *at the origin of life* directly challenges current paradigms of life’s emergence based on ‘from the simple to the complex’ – the complexification of matter [59]. The paradigm of complexification assumes that cellular building blocks (amino acids, nucleobases, sugars and their phosphate esters) and their polymers (proteins and nucleic acids) were ‘naturally synthesized’, i.e., non-enzymatically under ‘plausible’ prebiotic conditions, and then cells somehow ‘self-assembled’ by complexification and started to ‘live enzymatically’. Such a scenario represents a miracle of the skeptics’ camp of ‘almost miracles’ (Shapiro, personal communication, 2008). For the paradigm of ‘from the simple to the complex’ to succeed, an external agent needs be employed (Maxwell’s demon) that *selectively un-mixes complex mixtures of planetary prebiotic chemicals* for them to react in particular ways, which ignores the effects of the second law.

Complexification is contradicted by the results of Stanley Miller’s evolutionary (about a week long) experiments [60, 61], which gave a naturally complex mixture of products, the major ones being ‘intractable tars’ (oligomeric, polymeric and cross-linked), with small amounts of various small molecules including amino acids and other building blocks of life [12]. The role of tars in the origin of life has been neglected, even though they are surface active and likely to form the first complex ‘tarry vesicles’ [49] (David Deamer, personal communication, 2012). These tars have not been investigated in detail, particularly their surface-active and self-aggregating properties in hot electrolytes such as seawater. Nevertheless, such mixtures of complex carbon compounds, their low molecular weight precursors and hydrolytic byproducts, appear in the Universe, including the Murchinson meteorite [62–67]. Therefore, an alternative interpretation of Miller’s experimental paradigm suggests that the chemical evolution of microspaces began ‘proto-heterotrophically’ [68], in phase-separated multi-layered primordial analogs of extant lipid-protein bilayer membranes. These prebiotic amphiphilic phases were saturated with aqueous electrolytes and low molecular weight precursor molecules, such as amino acids and other life’s building blocks that were becoming available in the environment by non-enzymatic means as ‘true non-biological nutrients’.

At the bacterial end of the evolutionary continuum, the remnants of the complexity principle can be seen in bacterial energy utilization. That is, there are *many* bacterial red-ox chemistries (high chemical complexity) but just one chemical reduction practiced by highly evolved animals, the mitochondrial reduction of molecular oxygen to water (lower chemical complexity). Thus Earth’s evolution from ancestral prokaryotes to *Homo sapiens* is accompanied by the reduction of cellular *chemical* complexity and an increase in structural complexity (eukaryotic organelles, multicellularity). Such an increase does not seem to directly relate to increased genetic complexity, and the notion of evolutionary progress [toward perfection?] may need to be put to rest [69]. Thus, the nature of evolutionary complexity can be viewed as *chemical* (high at the origin of life and slowly decreasing on the evolutionary time scale), *genetic* (high and more or less constant since the origin of life, though debatable), and *structural* (increasing by rare large-scale, supramacromolecular fusions of living cells with each other and with fragments of dead organic and inorganic matter).

The fourth premise describes another aspect of complexity: the necessity of *crowding* of different molecules within microspaces, protocells, and contemporary cells, Figs. 1 and 3 [37, 41, 70–78]. Such crowding enables the evolution of molecular recognition and cytoplasmic self-organization. This is admittedly a chemical ‘reductionist’ premise, where natural recognition of cellular biomolecules, even as they are being synthesized (e.g., by ‘chaperone’ proteins), is necessary for the assembly and disassembly of multicomponent enzymes and cellular molecular machines (ATP-synthases, ribosomes, bacterial flagella, etc.), for enzyme-substrate interactions and for localizations of molecules within the cytoplasm. Molecular recognition and cellular self-organization are determined by non-covalent molecular forces and biochemical reactions within the cytoplasm [26, 41, 78]. The key ‘cytological’ forces are the hard-core excluded volume effect (size and shape of biomacromolecules), hydration for stabilization of biomacromolecular surfaces in aqueous media (hydrogen bonding), the hydrophobic effect (thermodynamically spontaneous separation and self-assembly of molecules that cannot hydrogen bond with water), and screened electrostatic attractions and repulsions between biomacromolecules with positive and negative charges mediated by aqueous electrolytes. The excluded volume effect at high ‘crowding’ ensures that the non-covalent forces act over a commensurate distance of about one nanometer [40, 41, 44, 45]. Thus chemical evolution of biomacromolecular surfaces under *molecular crowding* in confined microspaces in mixed electrolytes undergoing cyclic phase separations is a prerequisite for the transition from the inanimate to the living [26]. Only then could the evolution of proto-cytoplasmic molecules (molecular weight, homochirality, hydrophobicity, etc.) and their molecular self-organization (proto-metabolic and proto-genetic networks) proceed toward Darwinian thresholds.

Although crowding in bacterial cells is often pictured as ‘random packing’ [38, 79] with the nucleoid ‘floating’ in the middle, an alternative view pictures the cytoplasm as a ‘fast-growing and bustling well-organized city’ of biomacromolecular machines [80, 81]. The machines are wired by complex network of electrolyte pathways and stabilized by repulsive and attractive non-covalent forces, which brings about transient *non-random functional structuring* of the cytoplasm [41]. A coarse ‘chemical engineering’ model of such dynamic sol–gel structuring suggests that the vectorial biochemistry of the bilayer membrane [82, 83] extends (transiently) into the cytoplasm by 20-70 nm [46].

## Conclusions

Origins research exhibits an unusual degree of controversy because of the historical and evolutionary nature of the problem (You can’t run the tape backwards!), which limits the scope of experimentation and thus encourages speculation [9]. One example is the droll case of ‘directed panspermia’ in the camp of ‘almost miracles’ [84]. However, an additional reason for controversy is the lack of a good set of basic and uncontroversial premises to provide guidance for experimentation [60].

The premises described here address: (1) the continuity relation between chemical evolution and Darwinian evolution, (2) the planetary sources of energies necessary for chemical evolution, (3) the necessity for planetary physicochemical complexity at the origin of life, and (4) macromolecular crowding that ensures chemical evolution of molecular recognition and cellular self-organization. These premises lead to a new kind of non-equilibrium chemistry (see Box 1), i.e., *evolutionary* chemistry, which deals with supramacromolecular (‘cellular’) evolution of inanimate open thermodynamic systems, including their boundaries (‘membranes’), involving thousands of different molecules, driven by cyclic gradients of temperature, electromagnetic radiation and chemical potentials of environmental chemicals. Two kinds of complex inanimate systems have been proposed: (i) physical realization of chemical engineering simulators of historical inorganic (non-enzymatic) prebiotic world [32] to determine ‘what actually might have been going on’ during the Hadean eon, and (ii) experiments with dead populations of today’s bacteria to investigate the emergence of ‘being alive’ from biomolecules that once comprised a living state [33]. These experiments define a paradigm that addresses the compositions and processes that are critical for the conversion of complex non-equilibrium chemical states into living states. Current experimental paradigms for origins research based on ‘complexification of matter’ are untenable because they artificially eliminate chemical complexity and the thermal disordering effects of the second law of thermodynamics. Such laboratory procedures do not reflect Earth’s natural environments that gave rise to living states.

## Reviewers’ comments

### Review #1: Eugene V. Koonin, Senior Investigator, National Center for Biotechnology Information, National Institutes of Health, Bethesda, MD USA

The article by Spitzer et al., although not particularly lengthy, presents an extremely broad, panoramic outlook of the Origin of Life problem. To me, the key points are i) the continuity of chemical and biological evolution phases and ii) the importance of complex chemical (micro) environments and molecular crowding that in all likelihood were required to hatch life. I believe these points are valid and important. Certainly, they are not exactly new but at least the second one is often overlooked, so the present article will be a welcome addition to the literature. That said, perhaps not unexpectedly, given the scope of the article, I find certain gaps, omissions and logical inconsistencies in the presentation, as well as certain loose statements that are not directly relevant for the main theme of the article. I try to address some of these points below.

*We thank the reviewer for his positive comments regarding the distinction between chemical and biological evolution, and the importance of microenvironments and macromolecular crowding.*

* The authors repeatedly refer to the second law of thermodynamics and its “violations” by evolving complex systems, in some cases even making disingenuous statements such as “The paradigms of molecular replicators and self-organizing metabolic networks violate the second law of thermodynamics…” Certainly, they are well aware that the second law cannot be violated within its domain of applicability, i.e., in systems at equilibrium. However, this is not the type of thermodynamics that is important for understanding origin and evolution of life and complexity. Rather, these processes should be analyzed within the framework of non-equilibrium thermodynamics which indeed provides for self-organization and complexification. The authors do acknowledge this but only in passing and in rather vague terms. I am surprised that in a conceptual article like this, specific and relevant ideas from non-equilibrium thermodynamics, such as dissipative structures or self-organized criticality are not even mentioned. There is, obviously, literature on this subject, e.g., [1–4].

*We agree that ‘violation’ was too strong a description. The second law of thermodynamics represents the spontaneous flow of heat from hot to cold (redistribution of molecular energies that increases disorder). One consequence of the law is diffusion (entropic mixing), which eliminates concentration (and other) gradients, thus increasing disorder in multicomponent systems. Simply ignoring the 2*^*nd*^*law will not do, as diffusion is a prerequisite for life.*

*Life was hatched from multicomponent natural systems of molecules and, therefore, a mechanism is needed for selecting ‘life’s’ molecules (building blocks of amino acids, sugars and nucleobases and their phosphate esters and polymers) from all the other molecules. The models of replicators and metabolic cycles do not offer any such mechanism, in effect assuming their ‘miraculous’ and ‘separated’ appearance, essentially an action of Maxwell’s demon. We now describe the shortcomings of these ideas as ‘ignoring’ the second law.*

*We have addressed the possibility of cellular self-organization arising from Prigogine’s dissipative structures elsewhere (our reference* [26]*, pp. 375–6):*

“From a theoretical viewpoint, physicochemical gradients and their couplings offer a number of possibilities, starting from Onsager’s linear reciprocity relations for gradients and fluxes to the complexity theories of emergent phenomena motivated by Prigogine’s dissipative structures (41). In general, we can view the response of an equilibrium system to perturbing gradients as follows: (small) linear gradients from equilibrium give Onsager’s reciprocal relations and orderly steady states; larger departures from equilibrium lead to nonlinear responses, which may lead to chaotic behavior; and still larger departures may lead to the emergence of “energy-hungry”, self-organizing structures. Given the biological data on the precision of cell growth and division, on the various states of low metabolic activity or no metabolic activity (34), on the degree of homeostasis within the cell, and on the remarkable ability of a cell to resist injury and to self-repair (40), we doubt that life has much to do with the complexity of energy-dissipating structures at the edge of chaos (sensitivity to external initial conditions). Rather, the complexity seems to lie in the free-energy gradients of too many kinds of potential reactions and reaction mechanisms, which involve polymers (polyelectrolytes), ions, and molecules, and which are always coupled to external physical gradients through membranous walls. The biomacromolecular crowding leads to the emergent dynamic structuring of reaction pathways, as discussed later in the context of the concept of complex vectorial chemistry (112). Whether the self-organizing effects of dissipation of large amounts of metabolic energy (73, 74) cause the emergence of physical vectorial pathways remains to be determined”.

*We add that common and well-understood first and second order (but cyclic, our premise 2) phase transitions of physical chemistry (gas/vapor – liquid – solid; sol–gel transitions,* etc.*) are a simpler and better understood mechanism of creating order than ‘dissipating’ structures. The underlying reason for phase-separations (‘self-ordering’) are non-covalent molecular forces (our premise 4), which are also relatively well understood. However such classical phase transitions are poorly understood in multicomponent (complex) systems under cyclic evolutionary gradients that lead to dynamic pattern formation and dynamic ‘self-construction’ , our references* [1, 51]*. They can be studied experimentally, our references* [32, 33]*.*

One of the basic premises of the article is that “The fundamental question is whether ‘life as we know it and its astonishing chemical complexity is a very improbable occurrence or a nearly inevitable evolution of dynamic off-equilibrium chemical matter. The answers have been categorized into two ‘camps’– the camp of ‘almost miracles’ and the camp of laws of physics and chemistry.” I find this a false opposition and an apparent misunderstanding. Certainly, life evolved under the laws of physics – indeed, how can they be violated (in their appropriate domains of relevance). This indisputable fact in no way rules out the possibility that emergence of life is an extremely unlikely event and in that sense a “near miracle” (again, not in the sense that any laws break down).

*We have used Iris Fry’s designations (our reference 9) and Robert Shapiro’s elaborations (our reference 10) of ‘the camps’ as a description of the extreme views about the chances of life’s emergence. We assume near inevitability of life’s emergence, given the extant atomic composition of Earth (assumed similar to that of 3.5 billion years ago) and the resulting evolving chemistries during the first billion years since the formation of the Earth-Moon planetary system 4.5 billion years ago. It is from these chemistries that life ‘separated’ (our premise 3). The problem with the ‘near miracle’ assumption is that it invites considerations of ‘new principles’ that are unknown to physics and chemistry.*

I find the usage of “cell cycle” in this article to be rather disingenuous. For example, the following “Second, once cellular life is established, these gradients work on the cell cycle, bringing about cell cycle errors” is I think hardly a meaningful statement. And then, “The cell cycle errors result in phenotypic populations of ‘mutants’ (Darwinian variations)…” - strange and plainly incorrect.

The above is one example of the rather loose usage of biological concepts and terms. I think the article can benefit from careful editing aimed at removing such verbiage that potentially could detract from the important messages that authors are trying to convey.

1. Elia V, Germano R, Napoli E: Permanent Dissipative Structures in Water: the Matrix of Life? Experimental Evidences and their Quantum Origin. *Curr Top Med Chem* 2015.
2. Pulselli RM, Simoncini E, Tiezzi E: Self-organization in dissipative structures: a thermodynamic theory for the emergence of prebiotic cells and their epigenetic evolution. *Biosystems* 2009, 96 (3):237–241.
3. Bak P, Paczuski M: Complexity, contingency, and criticality. *Proc Natl Acad. Sci U S A* 1995, 92 (15):6689–6696.
4. Sneppen K, Bak P, Flyvbjerg H, Jensen MH: Evolution as a self-organized critical phenomenon. *Proc Natl Acad Sci U S A* 1995, 92 (11):5209–5213.

*Both reviewers comment that we are venturing too far into biological and philosophical aspects of the nature of life rather than its physicochemical emergence. We appreciate this and agree with their comments. We have removed the last two paragraphs.*

### Review #2: L. Aravind, Principal Investigator, National Center for Biotechnology Information, National Institutes of Health, Bethesda, MD USA

Spitzer et al. discuss the constraints from physical chemistry on the speculations regarding the origin of life. They suggest that application of these constraints helps design better empirical approaches to study the “pre-biotic” period of evolution.

On the whole the article is well-written and provides a reasonable review of the prior speculations and the state of our understanding of the origin problem. The emphasis on macromolecular crowding, hydration and the damped Coulomb interactions acting over a comparable distance and being critical in the pre-biotic self-construction and biochemistry is one of the key aspects of their proposal. This indeed cuts out certain classes of speculations and enforces the need for confined microspaces as a primary element in the origin of life. It also allows a degree of temperature independence for the origin of life.

*We thank the reviewer for the positive comments regarding the necessity to understand origin of life problems in terms of molecular forces.*

There are certain issues in the discussion that might lend themselves to alternative considerations:

* The authors following Woese propose that there was an initial phase with unstable membranes where lateral gene transfer was rampant. They posit that the Darwinian threshold is crossed only later when lateral transfer is diminished by stabilizing membranes. This point needs clarification because all empirical evidence from genomics suggests that lateral transfer is rather common, i.e., barring a relatively small core of ~50-60 genes the rest of the genes in prokaryotic genomes are routinely prone to lateral transfer and even that small core is not entirely immune. So what exactly is the threshold of lateral transfer they define for the transition to the so-called Darwinian threshold? Moreover, it is not as if prior to the surmised reduction in lateral transfer Darwinian evolution was not acting. We have much evidence that at least for the last ~2.4 billion years on earth lateral transfer has been spreading around “interesting” biochemical capabilities between distant organisms from a limited set of “nurseries” where they are invented (e.g., PMID: 24984775). This spread of biochemistry is operating under the Darwinian process of natural selection. So there is no reason that in the earlier phase, which the authors speculate about there, there was not a similar role for Darwinian process acting on the laterally transferred determinants of the biochemistry of the evolving transcription and translation apparatus. It is just that for these became more refractory to non-orthologous displacement in later biotic evolution.

*We share the concern of using the term ‘threshold’. The term implies relatively sudden transition between chemistry and biology. It is more likely that the transition was long and not very distinct (continuity premise 1), and therefore ‘threshold’ may not be the best term. This problem is analogous to determining the glass temperature of polymers, which can be a fairly sharp, but continuous 2nd order transition or a broad transition sometimes barely detectable in complex mixtures. In any case, the transition is a ‘historical’ one and its precise nature may remain unknowable. We do not use ‘threshold’ exactly in the same sense as Woese does, who assumes a fixed genetic code and subsequent evolution of the transcription/translational apparatuses. As per Fig.*1*, we indicate that proto membranes, proto nucleic acids (genetic code) and proto-proteins co-evolved chemically as a system of reaction patterns coupled and driven by the external gradient of temperature, water activity and electromagnetic radiation. We have now reversed the shading of the chemical evolution to be continuous with the biological evolution rather than indicating a ‘narrow’ transitional threshold.*

* The authors assume that the origin of life took place on earth. This assumes that all the prerequisites are found on typical rocky planets with iron cores. This is not entirely consistent with what we know thus far of rocky entities in planetary systems, especially in terms of the chemistry of tectonically active regions like those utilized by the authors in their proposal. How would concentrated lipids for example emerge in these zones? Moreover, the deep divergences of conserved systems between archaea and bacteria point to divergence times older than the earth’s age assuming they are evolving like other proteins. The “tars” which are mentioned in the text are more likely to have emerged on rocky moons of larger gaseous planets.

*We observe that Earth may be a rare (untypical) planet containing all the elements of the periodic table, and in proportions conducive for the chemical evolution of life’s building blocks and the eventual emergence of life. The quantum mechanical explanation of the periodic table tells us not to expect to discover any new elements of sufficient nuclear stability. Our point is that life cannot emerge without metals, which is an obvious point which we think is insufficiently appreciated, partly because the necessity for metals is not coded for in the genes, or only indirectly. It appears that water and ions represent a ‘relic’ of the environment from which life separated or arose,* e.g.*, cellular chemistry is based on potassium ion and other ions of relatively high ionic strength, and RNA does not work well without magnesium ion.*

* “With the appearance of three cellular designs – bacterial, archaeal and eukaryotic, Darwinian evolutionary processes then progressed by (i) micro-evolutionary errors in the genome, DNA chemical changes, and by (ii) macro-evolutionary (‘saltational’) rare events involving interactions of different cells and the morphogeneses of their membranes”

Lateral transfer, a major evolutionary process in the Darwinian realm, should be considered distinctly from the above two. The interactions between different cells, i.e., symbiosis, are common but symbiosis spawning a new major lineage of life namely eukaryotes was a one-off event but still part of the symbiosis continuum.

*We agree, and hope to elaborate this classification in the future. For now, being concerned with life’s emergence and less with its evolution*, *we include horizontal gene transfer in the macro-evolutionary category as a ‘catchall’ for any physicochemical process that involves ‘rupture and fusions’ of membranes, as in genetic engineering.*

*Heterotrophic origins of life: Entirely agree with that point but may want to credit

Sagan and Khare who had this idea long ago.

*We have included this very interesting reference in the revision.*

* The authors mention bacteria as the first cells: Should be more accurately the ancestral prokaryotic cells.

*We now make use of ‘ancestral’ in the revision. This is a very good point, which raises the question whether any of existing bacteria (some perhaps not yet discovered, or those that cannot be easily cultured) can be used as a meaningful ‘model’ for ancestral prokaryotic cells.*

* The authors state that the evolution of consciousness is a biological problem: This is stepping into really murky grounds as it raises a tricky philosophical question of consciousness as in qualia. It appears that the authors are conflating sensory signaling transduction and recording with consciousness.

*As noted in the reply to the first reviewer, we have removed the last two paragraphs to avoid philosophical contamination.*

Minor

* Formatting problems: some unformatted characters appear in the PDF as square boxes

*some references are un-inserted and have authors’ comments instead.

* “highly evolved animals”: animals are no less evolved than other extant life forms.

* “not-so-clever bacteria“: Was this tongue in cheek? Does not belong in a scientific paper

*Thank you for pointing out these errors. Yes compound adjective was used ‘tongue-in-cheek’. We have removed it.*

### Review #3: Doron Lancet, Crown Human Genome Center, Weizmann Institute of Science, Israel

This reviewer provided no comments for publication.

## Abbreviations

RNA: Ribonucleic acid
ATP: Adenosine triphosphate
